# Increased Production of IL-17A-Producing γδ T Cells in the Thymus of Filaggrin-Deficient Mice

**DOI:** 10.3389/fimmu.2018.00988

**Published:** 2018-05-08

**Authors:** Mia Hamilton Jee, Jeanne Duus Johansen, Terkild Brink Buus, Trine Hilkjær Petersen, Anne-Sofie Østergaard Gadsbøll, Anders Woetmann, Niels Ødum, Jacob Pontoppidan Thyssen, Andrea Jane White, Graham Anderson, Carsten Geisler, Charlotte Menné Bonefeld

**Affiliations:** ^1^Department of Immunology and Microbiology, Faculty of Health and Medical Sciences, University of Copenhagen, Copenhagen, Denmark; ^2^National Allergy Research Centre, Department of Dermato-Allergology, Copenhagen University Hospital Gentofte, Hellerup, Denmark; ^3^Institute of Immunology and Immunotherapy, University of Birmingham, Birmingham, United Kingdom

**Keywords:** *ft/ft* mice, filaggrin, γδ T cells, IL-17, thymus, development

## Abstract

Mutations in the filaggrin gene (*Flg*) are associated with increased systemic levels of Th17 cells and increased IL-17A production following antigen exposure in both humans and mice. In addition to Th17 cells, γδ T cells can produce IL-17A. The differentiation of γδ T cells to either IFNγ or IL-17A-producing (γδT17) cells is mainly determined in the thymus. Interestingly, it has been reported that filaggrin is expressed in the Hassall bodies in the human thymic medulla. However, whether filaggrin affects γδ T cell development is not known. Here, we show that filaggrin-deficient flaky tail (*ft/ft*) mice have an increased number of γδT17 cells in the spleen, epidermis, and thymus compared to wild-type (*WT*) mice. We demonstrate that filaggrin is expressed in the mouse thymic medulla and that blocking the egress of cells from the thymus results in accumulation of Vγ2^+^ γδT17 cells in the thymus of adult *ft/ft* mice. Finally, we find increased T cell receptor expression levels on γδ T cells and increased levels of IL-6 and IL-23 in the thymus of *ft/ft* mice. These findings demonstrate that filaggrin is expressed in the mouse thymic medulla and that production of Vγ2^+^ γδT17 cells is dysregulated in filaggrin-deficient *ft/ft* mice.

## Introduction

Filament aggregating protein (filaggrin) is a major structural skin protein that assists in the formation of the epidermal barrier. Filaggrin is a degradation product from the large pro-protein profilaggrin (>400 kDa). During terminal differentiation of keratinocytes, profilaggrin is carried apically, dephosphorylated and degraded into an N-terminal peptide, several filaggrin monomers and a C-terminal peptide (1, 2). The flaky tail (*ft/ft*) mouse is commonly used as a model of filaggrin deficiency exhibiting spontaneous development of eczematous skin lesions (3). The increased skin inflammation in filaggrin-deficient mice correlates with increased levels of IL-17A (4, 5). Accordingly, a strong reduction of skin inflammation is seen in IL-17A/filaggrin double-deficient mice compared to mice only deficient in filaggrin (6). In addition to the local IL-17A-driven skin inflammation, a systemic OVA-specific IL-17A response can be induced in filaggrin-deficient mice by exposure of the skin to OVA (3, 4). In line with this, we have recently shown that filaggrin deficiency is associated with an increase in the numbers of IL-17-producing T (Th17) cells in both humans and mice (7). Whether filaggrin deficiency affects other IL-17A-producing cell subsets is currently unknown.

γδ T cells can be divided into IFN-γ or IL-17-producers. The master transcriptional regulators of IFNγ and IL-17-producing γδ T (γδT17) cells are the T-box transcription factor (T-bet) and retinoic acid receptor-related orphan receptor-γt (RORgt), respectively (8). Whereas γδ T cells leave the thymus as naïve T cells and gain their effector function upon priming in peripheral lymphoid tissues, the effector fate of γδ T cells is mainly programmed in the thymus (9–12). The γδT17 cells can be divided into two groups: natural γδT17 cells, which are programmed for IL-17 production during their development in the thymus, and inducible γδT17 cells, which are primed for IL-17 production after leaving the thymus (9–13).

γδ T cells develop in distinct waves characterized by different Vγ segment usage (14). γδ T cells expressing Vγ1.1, Vγ3, or Vγ5 segments [Garman nomenclature (15)] primarily become IFN-γ producing cells, while γδ T cells expressing Vγ4 primarily become IL-17-producing cells. Interestingly, γδ T cells expressing the Vγ2 segment can develop into either IFNγ or IL-17-producing cells. Different signaling pathways determine whether a γδ T cell in the thymus will become IFNγ or IL-17 producing. Both T cell receptor (TCR)-dependent and TCR-independent signaling pathways are involved in γδ T cell development. Different subsets of thymic epithelial cells (TEC) provide the microenvironments needed for the development of T cells. Interestingly, it has been reported that terminally differentiated TEC in the Hassall’s corpuscles in the human thymic medulla express filaggrin. The important role of TEC in the development of conventional T cells is well described, but the role of TEC in the development of γδ T cells is less clear. However, it is believed that TEC provide distinct ligands or selecting molecules modulating the thymic programming of γδ T cells. Strong TCR signaling induces development of IFNγ producing cells, whereas missing or weak TCR signaling leads to development of γδT17 cells (9, 10, 16). In addition to TCR signaling, signaling *via* costimulatory receptors and cytokine receptors also affects γδ T cell development (11, 17–19). Signaling *via* CD27 seems to play an important role in the differentiation of γδ T cells in thymus as CD27^+^ γδ T cells differentiate into IFNγ-producing cells, whereas CD27^−^γδ T cells become IL-17 producing (11). Finally, the cytokine environment in the thymus regulates the differentiation of γδ T cells. TGFβ, IL-1, IL-23, and IL-6 seem to mediate the development of IL-17-producing γδ T cells (17).

In the present study, we investigated whether the production of γδ T cells is affected in filaggrin-deficient *ft/ft* mice. We found a fivefold increase of splenic and epidermal γδT17 cells in *ft/ft* mice compared to wild-type (WT) mice. This increase of γδT17 cells was associated with an enhanced production of γδT17 cells in the thymus. In addition, we found that filaggrin is expressed in the thymus medulla of WT mice and that filaggrin expression is reduced in the thymus of *ft/ft* mice. Further analyses showed that the increased number of γδT17 cells was primarily contained within the Vγ2^+^ subset. Finally, we found higher TCR expression levels on γδ thymocytes and higher levels of IL-6 and IL-23 in the thymus of *ft/ft* mice compared to *WT* mice.

## Materials and Methods

### Animal Model

Flaky tail mice (*a/a Tmem79^ma^ Flg^ft^/J*, stock number 000281) (*ft/ft*) were purchased as cryopreserved embryos from the Jackson Laboratory and bred at our in-house animal facility. Age-matched, mixed gender C57Bl/6 mice were purchased from Janvier or Taconic Laboratories. Experiments were performed on the mice at the age of 8–12 weeks. The mice were housed in the specific pathogen free animal facility at the Department of Experimental Medicine, Panum Institute, in accordance with the national animal protection guidelines (license number 2012-15-2934-00663). C57Bl/6 mice were used as WT controls as *ft/ft* mice have previously been described to be outcrossed onto C57Bl/6 mice. However, *ft/ft* is not a strict congenic strain, but a semi-inbred strain (5). In some experiments, mice were treated with FTY720 (2.5 µg/ml) in their drinking water for six consecutive days.

### Preparation of Single-Cell Suspensions

Single-cell suspensions from thymi, lymph nodes, and spleens were prepared by dissociating the organs on 70 µm cell strainers. The single cells were washed in RPMI medium (10% FBS, 0.5 IU/L penicillin, 500 mg/L streptomycin, 1% l-glutamine), and cell suspensions were adjusted to 2 × 10^7^ cells/mL. Subsequently, 100 μL/well was plated in a round-bottomed 96-well plate. Single-cell suspensions from the epidermis were isolated from the ears. The ears were split into a dorsal and ventral part. The dorsal part was transferred to a 0.3% trypsin-GNK (2.94 g NaCL, 0.134 g KCl, 0.334 g glucose/dextrose per 1 g of trypsin) solution for 60 min at 37°C, 5% CO_2_ with the dermis side down. The epidermis was peeled from the dermis and transferred to 0.3% trypsin-GNK with 0.1% DNase and left at 37°C for 10 min. Cells were filtered through a cell strainer, washed and plated overnight at 37°C, 5% CO_2_ to allow re-expression of surface markers.

### Staining and Flow Cytometry

Fc-receptors were blocked with anti-CD16/CD32. Surface markers on cells were stained with anti-CD3ε, -TCRγδ(GL3), -CD4, -CD8α, -CD24, -CD25, -CD44, -CD27, CD45RB, -CCR6, -Vγ1, -Vγ2, and -Vγ3 diluted in Brilliant Stain Buffer (BD Biosciences). Viability of cells was determined using Fixable Viability Dye (eFlour^®^ 780) (eBioscience). When staining for intracellular cytokines, the cells were first stimulated with PMA (50 ng/ml), monensin sodium (4 µg/ml), and ionomycin (500 ng/ml) for 4 h and stained for surface markers. Following fixation and permeabilization with BD Cytofix/Cytoperm (BD Biosciences), the cells were stained for intracellular cytokines with anti-IL-17A and anti-IFNγ antibodies. Data were collected on a BD LSRFortessa and analyzed with FlowJo Software.

### Histology and Staining for Confocal Microscopy

Ears and thymi from *ft/ft* and C57Bl/6 mice were transferred to formaldehyde. Histology was performed by Nordic Biosite, Finland. Sections were stained with hematoxylin and eosin and with antibodies targeting filaggrin (Poly19058, BioLegend).

For confocal microscopy analyses, fresh thymi were imbedded in OCT compound (Sakura Fintek) and snap frozen on dry ice. The tissue was cut into 7 µm sections and fixed in acetone. The following antibodies were used for staining: rabbit anti-filaggrin (Poly19058, BioLegend), AlexaFluor 647 anti-mouse CD4 (GK1.5, BioLegend), and biotinylated anti-mouse CD8a (53-6.7, eBioscience). To detect the anti-filaggrin antibody, an AlexaFluor 555 donkey anti-rabbit IgG (Invitrogen) antibody was used. Biotinylated CD8 antibody was detected with Streptavidin conjugated to AlexaFluor 488 (Life Technologies). Purified rabbit polyclonal isotype control (Poly19058, Biolegend) was used as control to filaggrin stains. Sections were analyzed using a Zeiss LSM 880 confocal microscope.

### Quantitative Real-Time PCR

Organs frozen in liquid nitrogen were disintegrated in a Precellys tissue homogenizer (Bertin Technologies) in 500–1,000 mL of TRI Reagent (Sigma Aldrich). For RNA extraction from thymic stromal cells (TSC), fresh thymi were cut into 6–8 pieces and thymocytes mechanically released by pipetting and changing of medium, and finally disintegrated as described above. Following centrifugation, the supernatant was mixed with 1-Bromo-3-chloropropane (Sigma Aldrich), samples were centrifuged and the upper phase recovered. RNA isolation was performed using the RNeasy Mini Kit 250 (Qiagen) according to manufacturer’s specifications. RNA concentrations were measured using a Nanodrop 2000c spectrophotometer (Thermo Scientific), and RNA was diluted to a final concentration of 2 µg/µL. RNA was transcribed into cDNA using the RevertAid First Strand cDNA Synthesis Kit (Fermentas). Transcription of genes was measured by real-time PCR. Stock Taqman primer/probe sets with Taqman Universal Master Mix was processed in a Stratagene Mx3000P/Mx3005P (AH Diagnostics/Agilent Technologies), and the data was analyzed using MxPro software. Transcription of target genes was calculated relatively to GAPDH.

### Protein Extraction

Extraction of protein was performed by lysing ears with lysis buffer (50 mM Tris Base, 250 mM NaCl, 5 mM EDTA, 1% Triton X-100), and disintegrating the samples on a Precellys tissue homogenizer (Bertin Technologies). Subsequently, samples were spun down, and the supernatant was recovered. To purify TSC, thymi were cut into 6–8 pieces, and the thymocytes were mechanically released by pipetting up and down with a 1,000 µl pipette tip with the outermost end trimmed off. Following removal of media containing the thymocytes, fresh media was added and the release of thymocytes was repeated two times. TSC were lysed and protein extracted as described above.

### ELISA

Protein lysates were adjusted to a concentration of 3.0 µg/µl following determination of concentration by Bradford assay. Concentrations of IL-6 and IL-23 were determined using Mouse IL-6 ELISA Ready-SET-Go and mouse IL-23 ELISA Ready-SET-Go kits (eBioscience) according to manufacturer’s specifications.

### Statistical Analysis

Differences between groups were evaluated by the two-tailed unpaired Student’s *t*-test. The statistical analysis was performed using GraphPad Prism version 6.0, and a *p*-value below 0.5 was considered statistically significant. Statistical significance *p*-values are denoted as: *<0.05, **<0.01, ***<0.001, ****<0.0001.

## Results

### *ft/ft* Mice Have Increased Numbers of γδT17 Cells in the Spleen and Epidermis

**Figure 1:**
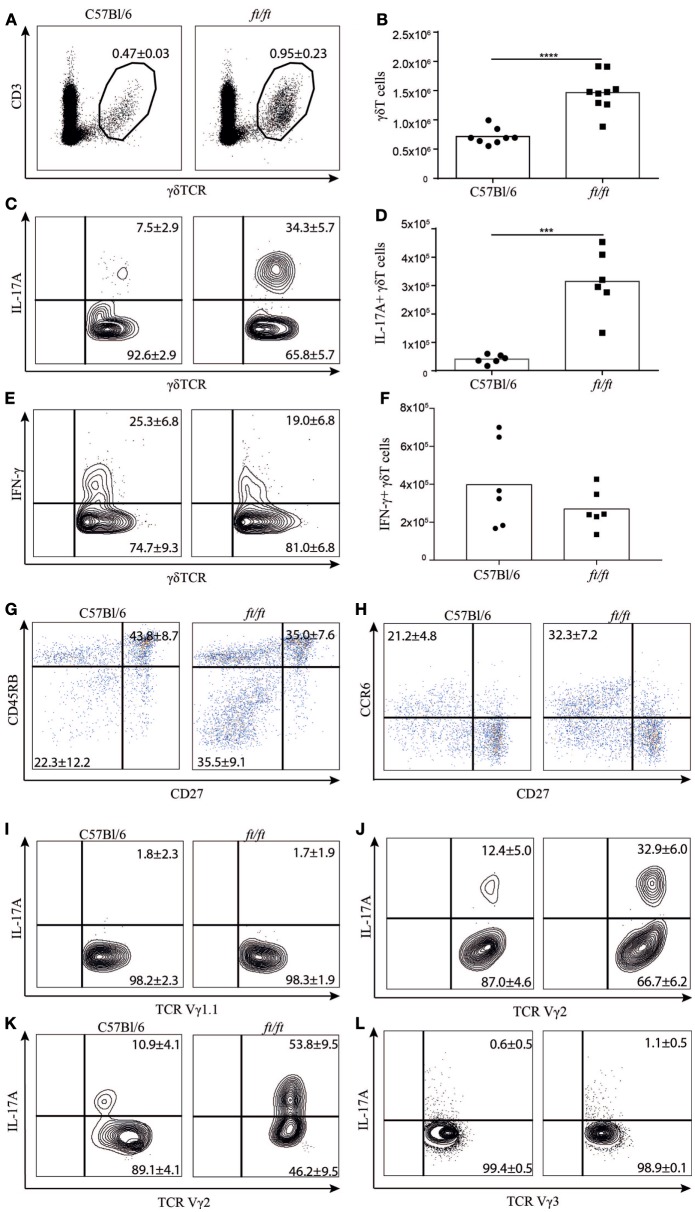
Increased numbers of γδT17 cells in the spleen and epidermis of *ft/ft* mice. **(A–J)** Flow cytometric analyses of spleen cells from 8 to 12 weeks old C57Bl/6 and *ft/ft* mice. **(A)** Fraction of CD3ε^+^TCRγδ^+^ spleen cells. **(B)** Absolute numbers of CD3ε^+^TCRγδ^+^ spleen cells. **(C–F)** Fraction and absolute numbers of **(C,D)** IL-17^+^ and **(E,F)** IFN-γ^+^ TCRγδ^+^ cells. **(G,H)** Dot plots showing CD27 versus CD45RB and CD27 versus CCR6 expression of TCRγδ^+^ cells. **(I,J)** Plots showing IL-17 expression on TCR Vγ1.1^+^ or TCR Vγ2^+^. **(K,L)** Plots showing IL-17 expression on TCR Vγ2^+^ and TCR Vγ3^+^ cells from the epidermis. Data are representative of two to three independent experiments with three to four mice in each. The mean percentages ± SD are given for the relevant populations in the plots.

To investigate whether γδ T cells might be involved in the IL-17 driven immune responses in *ft/ft* mice, we examined the distribution of γδ T cells in the spleen of *ft/ft* and WT (C57Bl/6) mice. The percentages as well as the absolute numbers of γδ T cells were significantly increased in *ft/ft* mice compared to WT mice (Figures 1A,B). Next, we investigated whether the increase of γδ T cells in the spleen of *ft/ft* mice also resulted in an increased number of γδT17 cells by determining the number of γδ T cells expressing IL-17A. We found significantly increased percentages and absolute numbers of γδ T cells expressing IL-17A in the spleen of *ft/ft* mice compared to WT mice (Figures 1C,D). Interestingly, this increase was specific for IL-17A-producing cells as no significant differences were seen in the percentages or numbers of IFN-γ-producing γδ T cells between *ft/ft* and WT mice (Figures 1E,F). The elevated number of IL-17A-producing cells seen in *ft/ft* mice seemed to be specific for γδ T cells and CD4^+^ T cells, as no differences in the frequencies of IL-17A-producing CD8^+^ T cells or non-T cells were observed between *ft/ft* and *WT* mice (Figures S1–S3 in Supplementary Material). Expression of CD27, CD45RB, and CCR6 can be used to determine γδ T cell subsets that produce IL-17A or IFN-γ (11, 13, 20). We found significantly increased fractions of CD27^−^CD45RB^−^ or CD27^−^CCR6^+^ splenic γδ T cells in *ft/ft* mice (Figures 1G,H) in agreement with the observation described above. To determine if the accumulating γδT17 cells were restricted to a specific subset of γδ T cells in the spleen of *ft/ft* mice, we co-stained for IL-17A and Vγ1.1 or Vγ2. The increased fraction of IL-17A^+^ cells seemed to be restricted to the Vγ2^+^ subset (Figures 1I,J). As *ft/ft* mice develop spontaneous skin inflammation (3), we next wanted to determine if elevated numbers of γδT17 cell also were found in the epidermis of *ft/ft* mice. We found a highly increased fraction of Vγ2^+^ T cells as well as an increased fraction of Vγ3^+^ T cells, the major T cell subset within epidermis, that were IL-17A^+^ in the epidermis of *ft/ft* mice compared to *WT* mice (Figures 1K,L). Taken together, these data indicated that the peripheral γδ T cell population, including γδT17 cells, is significantly expanded in *ft/ft* mice compared to *WT* mice.

### *ft/ft* Mice Have Increased Numbers of γδT17 Cells in the Thymus

**Figure 2:**
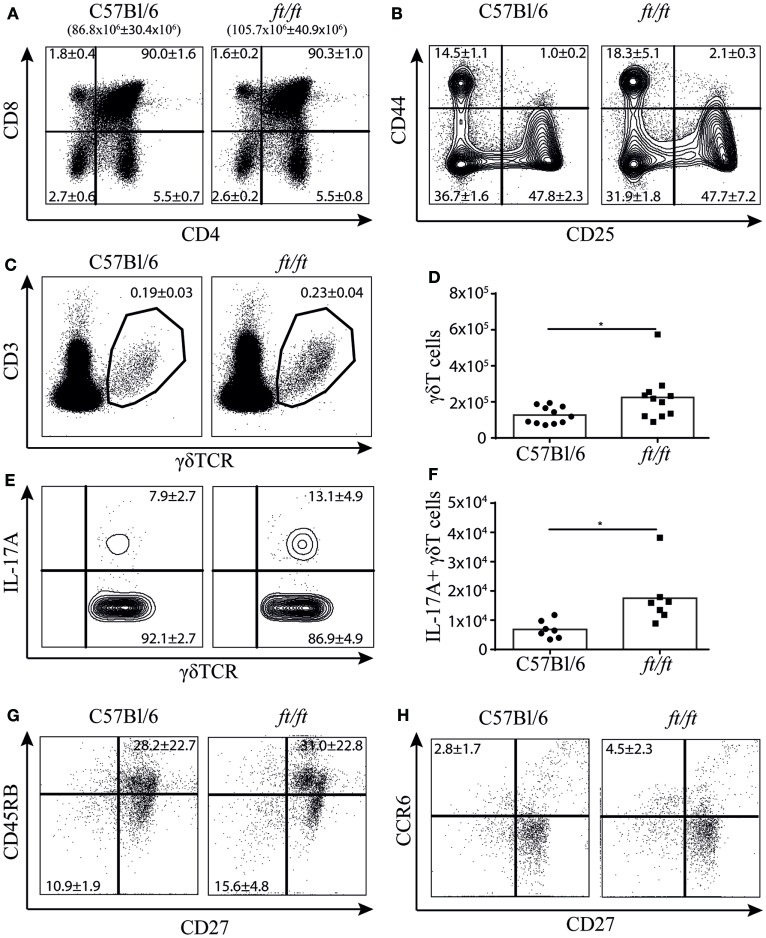
Increased numbers of γδT17 cells in the thymus of *ft/ft* mice. **(A–H)** Flow cytometric analyses of thymocytes from 8 to 12 weeks old C57Bl/6 and *ft/ft* mice. **(A)** Plots showing CD4 and CD8 staining of total thymocytes. The total numbers of thymocytes are given above the plots. **(B)** Plots showing CD25 and CD44 staining on the double negative CD4^−^CD8^−^ thymocyte population. **(C,D)** Fraction and absolute numbers of CD3ε^+^TCRγδ^+^ thymocytes. **(E,F)** Fraction and absolute numbers of IL-17^+^ TCRγδ^+^ thymocytes. **(G,H)** Dot plots showing CD27 versus CD45RB and CD27 versus CCR6 expression of TCRγδ^+^ thymocytes. Data are representative of two to four independent experiments with two to four mice in each. The mean percentages ± SD are given for the relevant populations in the plots.

To establish whether the increased number of γδT17 cells found in the peripheral lymphoid organs of *ft/ft* mice originated from natural γδT17 cells programmed in the thymus, we investigated the cellular distribution in the thymus of *ft/ft* and *WT* mice. We found no significant differences between *ft/ft* and *WT* mice in the fraction of double positive (CD4^+^CD8^+^), CD4 single positive and CD8 single positive cells or in the double negative (CD4^−^CD8^−^) 1–4 fractions (CD44^+^CD25^−^, CD44^+^CD25^+^, CD44^−^CD25^+^ and CD44^−^CD25^+^, respectively) (Figures 2A,B). However, *ft/ft* mice on average had a 20% increase in their total numbers of thymocytes as compared to *WT* mice (Figure 2A). Next, we analyzed the γδ T cell populations. We observed a significantly larger population of γδ T cells in the thymi of *ft/ft* mice compared to WT mice, which primarily was caused by the general increase in cell numbers in the thymi of *ft/ft* mice compared to WT mice (Figures 2C,D). Despite the similar fraction of total γδ T cells, we found a significant increase in both the fraction and the total numbers of γδ T cells expressing IL-17A in *ft/ft* compared to *WT* mice (Figures 2E,F). In accordance with this, we found a significant increase in the fraction of CD27^−^CD45RB^−^ (Figure 2G) and CD27^−^CCR6^+^ γδ T cells (Figure 2H) in *ft/ft* compared to *WT* mice.

### Filaggrin Is Expressed in the Thymic Medulla of Mice

**Figure 3:**
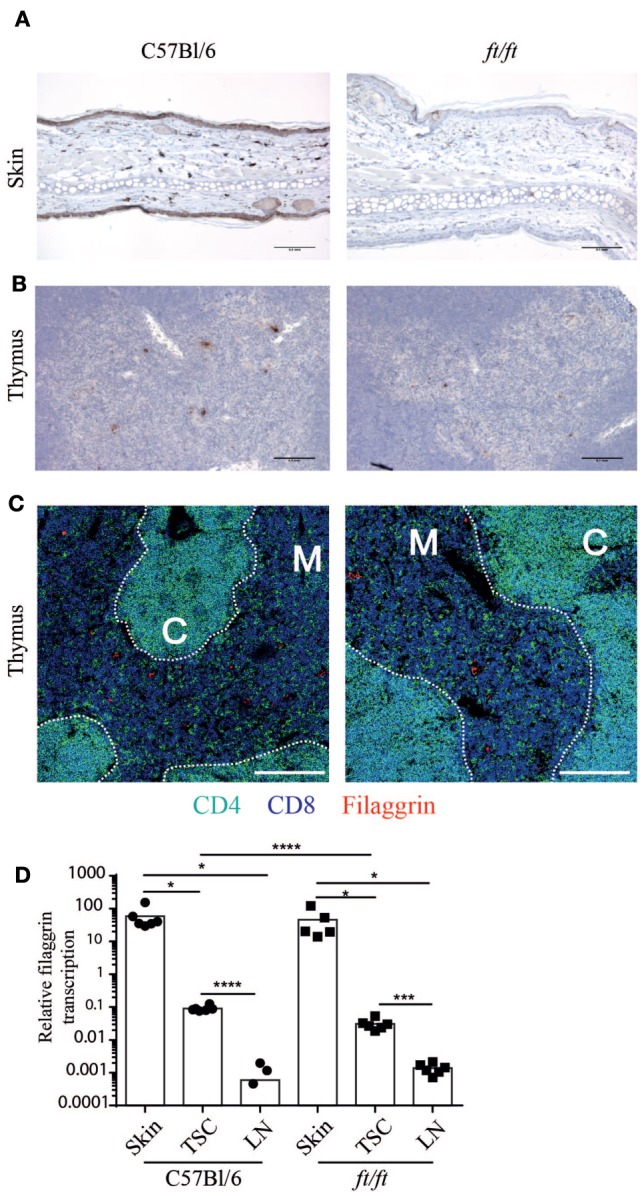
Decreased filaggrin expression at the transcriptional level in the thymus of *ft/ft* mice. **(A–C)** Immunohistological analyses of ears and thymi from 8 to 12 weeks old C57Bl/6 and *ft/ft* mice. Sections of **(A)** ears and **(B)** thymi were stained with anti-filaggrin antibody. The sections are magnified ×200 and the bars indicate 0.1 mm. **(C)** Confocal microscopy images of thymi showing expression of CD4 (green), CD8 (blue), and filaggrin (red). The bars indicate 200 µm. **(D)** Relative profilaggrin transcription in the skin, thymic stromal cells (TSC), and lymph nodes of *ft/ft* and C57Bl/6 mice. The Ct-values of profilaggrin transcription were normalized to the transcription of GAPDH. The means (*n* = 6) are indicated by bars. For C57Bl/6 lymph nodes, three values of profilaggrin transcription were zero and are therefore not indicated with dots on the logarithmic scale. Expression of profilaggrin of TSC was compared to skin and lymph nodes for each group.

Next, we speculated whether filaggrin is expressed in the thymus of mice and thereby could affect γδ T cell development. In humans, filaggrin is expressed in the Hassall’s corpuscles (21, 22), but it is unknown whether filaggrin is expressed in the thymus of mice. To determine the expression and location of filaggrin in the thymus, we compared thymi of *ft/ft* mice and WT mice using immunohistochemistry. Skin was used as a positive control (Figure 3A). Interestingly, we found that filaggrin is expressed in small clusters of cells in the thymic medulla in WT mice, and to a lesser extend in the thymic medulla of *ft/ft* mice (Figures 3B,C). The *ft* mutation carried by the *ft/ft* mice is a frameshift mutation that results in the expression of a truncated profilaggrin and almost complete absence of filaggrin monomers in the epidermis of *ft/ft* mice (3). Thus, the mutation does not necessarily cause a decreased transcription of *Flg*. We found that filaggrin is transcribed in thymic stroma of both *ft/ft* and *WT* mice, but to a lesser degree than seen in skin (Figure 3D). Furthermore, we found an approximately threefold reduction in the transcription of filaggrin in the TSC of *ft/ft* mice compared to *WT* mice (Figure 3D). Taken together, these data show that filaggrin is expressed at the protein and RNA level in the thymic medulla of *WT* and *ft/ft* mice and that the expression is lower in *ft/ft* mice.

### γδT17 Cells Continue to Be Produced After Birth in the Thymus of *ft/ft* Mice

**Figure 4:**
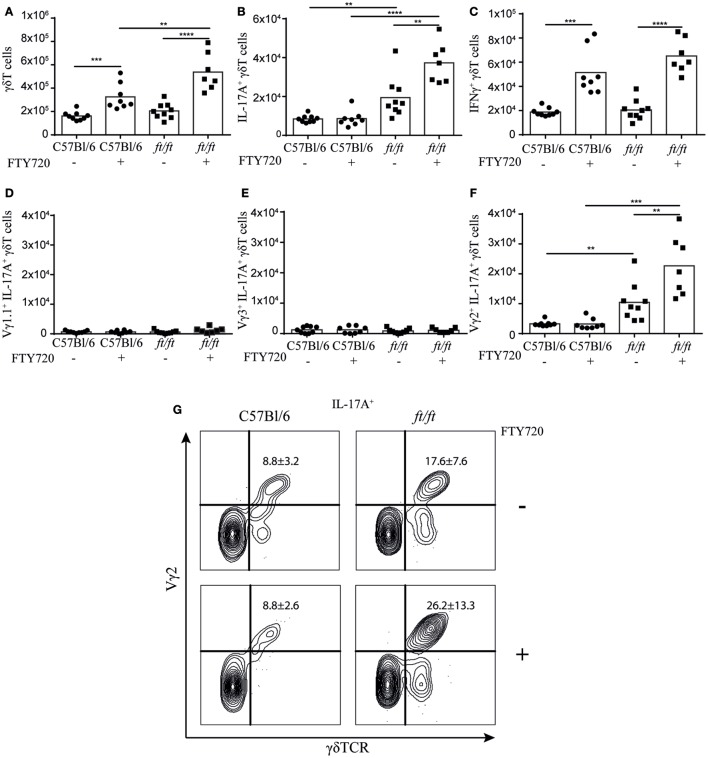
*ft/ft* mice accumulate γδT17 cells in the adult thymus. Numbers of γδTCR cells and γδTCR subsets in the thymus of 8–12 weeks old *ft/ft* and C57BL/6 mice treated with FTY720 or untreated. Single-cell suspensions were stained with anti-TCRγδ, -TCRβ, -IL-17A, –IFN-γ. -Vγ1.1, -Vγ2, and -Vγ3. Percentages of each population were multiplied with the cell count of the parent gate to get the numbers presented. **(A)** Numbers of γδTCR cells. **(B)** Numbers of IL-17A^+^ γδTCR cells. **(C)** Numbers of IFN-γ^+^ γδTCR cells. **(D)** Numbers of Vγ1.1^+^IL-17A^+^ γδTCR cells. **(E)** Numbers of Vγ3^+^IL-17A^+^ γδTCR cells. **(F,G)** Numbers and percentage of Vγ2^+^IL-17A^+^ γδTCR cells.

It has been described that natural γδT17 cells normally are produced only during fetal stages (12, 23). To determine whether the increase of γδT17 cells in the thymus of *ft/ft* mice was a reminiscence from the fetal stage or was due to an ongoing development during adulthood, we analyzed mice treated with FTY720, an inhibitor of S1P-R1-mediated thymic egress (24). The total numbers of γδ T cells were significantly higher in both *ft/ft* and *WT* thymus following FTY720 treatment. However, in *ft/ft* mice, the accumulation of γδ T cells was significantly greater than in WT mice (Figure 4A). Consistent with previous studies (12, 23), adult *WT* mice did not accumulate γδT17 cells following FTY720 treatment (Figure 4B). However, in contrast to *WT* mice, we found that *ft/ft* mice accumulated γδT17 cells in the thymus when treated with FTY720 (Figure 4B). This difference was specific to γδT17 cells as IFNγ^+^ γδ T cells accumulated to the same degree in *ft/ft* and *WT* mice (Figure 4C). To determine if the accumulating γδT17 cells were restricted to a specific subset of γδ T cells in the *ft/ft* thymi, we co-stained with Vγ1.1, Vγ2, and Vγ3. No significant accumulation of Vγ1.1^+^ or Vγ3^+^ γδT17 cells was detected (Figures 4D,E), whereas Vγ2^+^ γδT17 cells accumulated significantly (Figures 4F,G). As the accumulation of Vγ2^+^ γδT17 cells only accounted for approximately 50% of the total number of accumulated γδT17 cells in the thymus of *ft/ft* mice other γδT cell subsets, most likely Vγ4^+^ γδT17, probably also accumulated. Taken together, these experiments indicated that production of Vγ2^+^ γδT17 cells is dysregulated in *ft/ft* mice and that Vγ2^+^ γδT17 cells continue to be produced in the thymus of adult *ft/ft* mice.

### Increased Levels of IL-6 and IL-23 in the Thymus of *ft/ft* Mice

**Figure 5:**
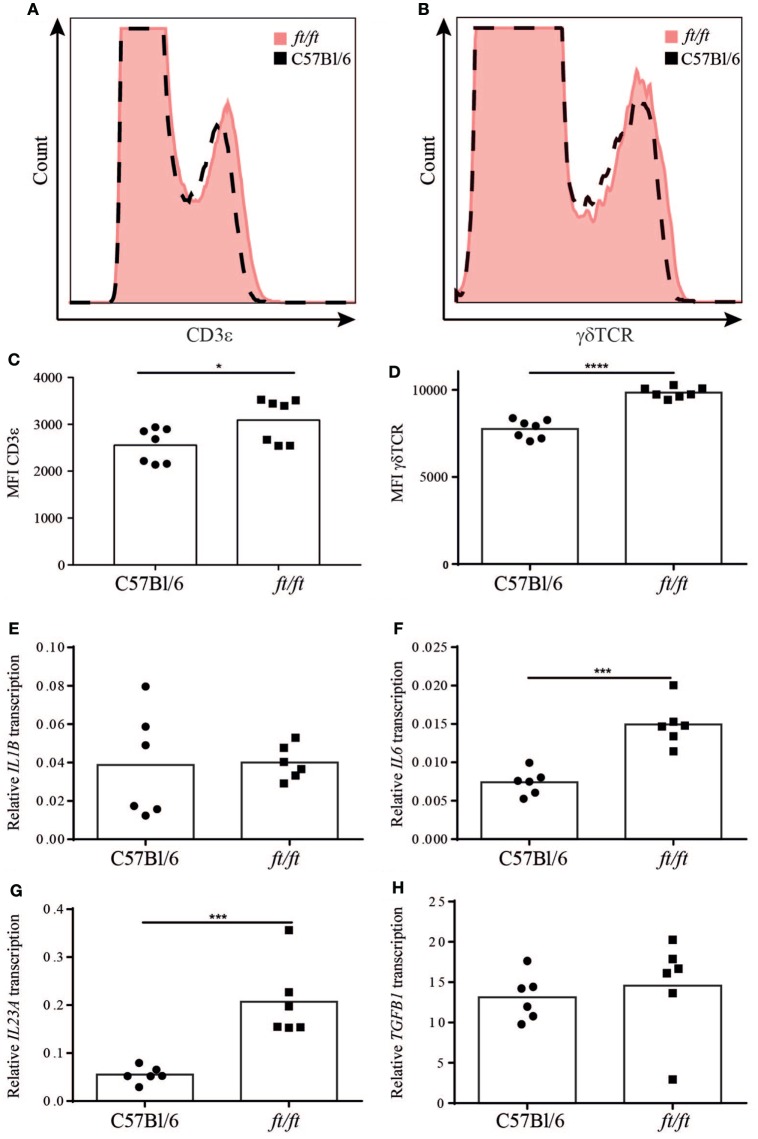

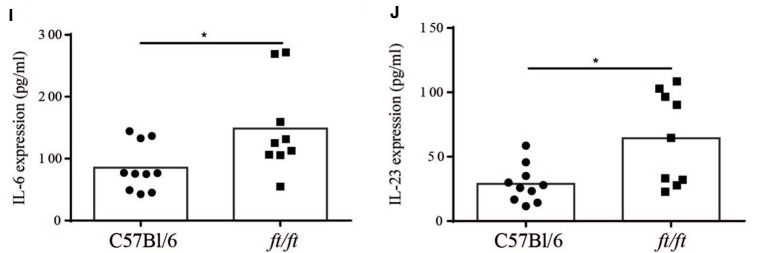
Altered TCR expression and cytokine expression in *ft/ft* mice. Representative histograms of **(A)** CD3ε and **(B)** TCRγδ expression on thymocytes from 8 to 12 weeks old wild-type *(WT)* (dashed lines) and *ft/ft* (red filling) mice. Mean fluorescence intensity (MFI) of **(C)** CD3ε and **(D)** γδTCR expression. **(E–H)** Relative gene expression of *IL1B, IL6, IL23*, and *TGFB1* in the thymi from *ft/ft* and *WT* mice. The Ct-values of the specific gene transcription were normalized to the transcription of GAPDH. **(I,J)** Concentrations of IL-6 and IL-23 in thymic lysates from *ft/ft* and *WT* mice.

The exact mechanisms determining the effector fate of γδ T cells in the thymus have yet to be fully uncovered, but antigen-naïve γδ T cells have been shown to produce IL-17, whereas antigen-experienced γδ T cells produce IFNγ (10). As the TCR expression level has been suggested to be a marker for whether the γδ T cells have encountered antigen or not (10), we determined the TCR expression levels on thymic γδ T cells from *ft/ft* and *WT* mice. In accordance, with the increased number of γδT17 T cells, we found higher expression levels of both TCRγδ and CD3εon thymic γδ T cells in *ft/ft* mice compared to *WT* mice (Figures 5A–D). To further investigate possible mechanisms mediating the increased development of γδT17 cells in *ft/ft* mice, we analyzed the expression of factors known to be involved in the differentiation of γδ T cells and Th17 cells. Interestingly, we found a significantly increased transcription of IL6 and *IL23A*, but not of *IL1B* and *TGFB1* in *ft/ft* mice compared to *WT* (Figures 5E–H). Accordingly, we found significantly higher protein levels of IL-6 and IL-23 in *ft/ft* mice compared to *WT* (Figures 5I,J).

## Discussion

In this study, we show that adult *ft/ft* mice have an increased number of γδT17 cells in the thymus, spleen and epidermis compared to WT mice. Furthermore, we demonstrate that filaggrin is expressed by TSC in the thymic medulla of *WT* mice and that this expression is decreased at both the transcriptional and translational level in the thymus of *ft/ft* mice. Blocking thymic egress resulted in an accumulation of Vγ2^+^ γδT17 cells in *ft/ft* mice, which was not seen in *WT* mice. Finally, we found an increased TCR expression level on thymic γδ T cells and an increased level of IL-6 and IL-23 in the thymi of *ft/ft* mice compared to *WT* mice.

The spontaneous skin inflammation found in *ft/ft* mice correlates with increased levels of IL-17A in the skin (4). In agreement with this, we found increased numbers of γδT17 cells in the epidermis of *ft/ft* mice compared to *WT* mice. Interestingly, we found that the majority of γδT17 cells in epidermis of *ft/ft* mice belonged to the Vγ2 subset, which are normally not present in the epidermis. In addition to the mutation in the *Flg* gene, *ft/ft* mice also have a mutation in the *Tmem79* gene. Therefore, we cannot exclude the possibility that the *Tmem79* gene plays a role in the increased number of γδT17 cells in the *ft/ft* mice. However, an increased level of IL-17A has been found in the skin of pure filaggrin-deficient mice supporting the importance of the Flg gene in γδT17 cell homeostasis (25).

Thymic crosstalk is the term used to describe the bidirectional need of TEC for development of T cells and of T cells for the development of TEC. In *WT* mice, γδT17 cells are only produced in the fetal thymus (12). In contrast, we show that γδT17 cells still are produced in the thymus of adult *ft/ft* mice. The mechanisms behind this are still unclear. However, distinct programs of thymus epithelial cell development exist in the fetal and adult thymus. Thus, the ability of the adult *ft/ft* thymus to continue to produce a γδ T cell subset typical of the embryonic thymus could indicate that the switch from fetal to adult programmes of TEC development are disturbed in *ft/ft* mice. Relevant to this, filaggrin expression in human thymus maps to Hassall’s corpuscles, a product of mTEC terminal differentiation that is first evident in mice after birth (26). Thus, filaggrin may be required for an mTEC terminal differentiation programme that marks age-related changes in the thymic microenvironment, which then controls the ability of the thymus to support different programs of T cell development at specific developmental stages.

The γδTCR signaling strength determines which effector subset the thymic γδ T cells will commit to; strong γδTCR signaling results in development of IFN-γ producing cells and weak signaling in IL-17A producing cells (9–11, 16). A central regulator of TCR signaling in thymic γδ T cells is the TCR expression level (27). Mice with reduced TCR expression level on their thymic γδ T cells have reduced TCR signaling and increased development of γδT17 cells compared to WT mice (27). Based on this, one could suspect that the increased TCR expression level we find on thymic γδ T cells in the *ft/ft* mice compared to WT mice would result in an increased generation of IFN-γ producing γδ T cells. In contrast, we found increased levels of γδT17 cells in both the thymus and periphery of *ft/ft* mice. However, increased TCR expression levels are found on thymic γδT cells in mice where the TCR ligand is not expressed compared to mice where the TCR ligand is expressed and lack of TCR ligand expression correlated with increased development of γδT17 cells (10). Furthermore, it is well described that T cells down-regulate the TCR on their surface as part of T cell activation (28). It is therefore possible that the increased TCR expression found on thymic γδ T cells in the *ft/ft* mice is due to reduced expression of TCR ligand in these mice. Although our observations indicate that the development of Vγ2^+^ γδT17 cells is dysregulated in *ft/ft* mice, we cannot formally exclude that the increased numbers and accumulation of Vγ2^+^ γδT17 cells are caused by an increased thymic expansion of mature Vγ2^+^ γδT17 cells in *ft/ft* mice that would normally be restricted in some way by filaggrin in WT mice. However, we could conclude that the production of Vγ2^+^ γδT17 cells in the thymus of *ft/ft* mice is increased.

In conclusion, in this study, we establish that filaggrin is expressed in the thymic medulla of *WT* mice, and this expression is decreased at both the transcriptional and translational level in *ft/ft* mice. Furthermore, we show that there is an enhanced production Vγ2^+^ γδT17 cells in the thymus of filaggrin-deficient mice and that there is a general increase in the number of thymocytes. Therefore, we suggest that reduced expression of filaggrin in the thymus affects the production of γδ T cells, which leads to increased IL-17 polarization. Currently, mutations in *Flg* are primarily associated with skin disease, but our results support that they might also cause systemic alterations in the immune system. This is supported by the observation that both humans and mice with filaggrin deficiency have systemically elevated levels of Th17 cells. As 8–10% of the European population are carriers of a filaggrin mutation, it is important to uncover still unknown effects of this mutation, and it will be very interesting to analyze whether humans with filaggrin deficiency have elevated numbers of γδT17 cells and if so whether they represent natural or inducible γδT17 cells.

## Ethics Statement

The mice were housed in the specific pathogen free animal facility at the Department of Experimental Medicine, Panum Institute, in accordance with the national animal protection guidelines (license number 2012-15-2934-00663).

## Author Contributions

MJ, CB, AG, AW, and TP performed the laboratory experiments. MJ, JJ, TB, AW, NØ, JT, AW, GA, CG, and CB conceived and designed the experiments. MJ, CG, and CB analyzed the data and wrote the paper. All authors revised and approved the final manuscript.

## Conflict of Interest Statement

The authors declare that the research was conducted in the absence of any commercial or financial relationships that could be construed as a potential conflict of interest.

## Abbreviations

DETC, dendritic epidermal T cells; Flg, filaggrin gene; ft, flaky tail; TCR, T cell receptor; TEC, thymic epithelial cells; TSC, thymic stromal cells; WT, wild type; γδT17, 17-producing γδ T cells.
