# Supplementary figures and images for: Increased Production of IL-17A-Producing γδ T Cells in the Thymus of Filaggrin-Deficient Mice
Source: Front Immunol. 2018 May 8;9:988. doi: 10.3389/fimmu.2018.00988 (PMC5953325; doi:10.3389/fimmu.2018.00988)

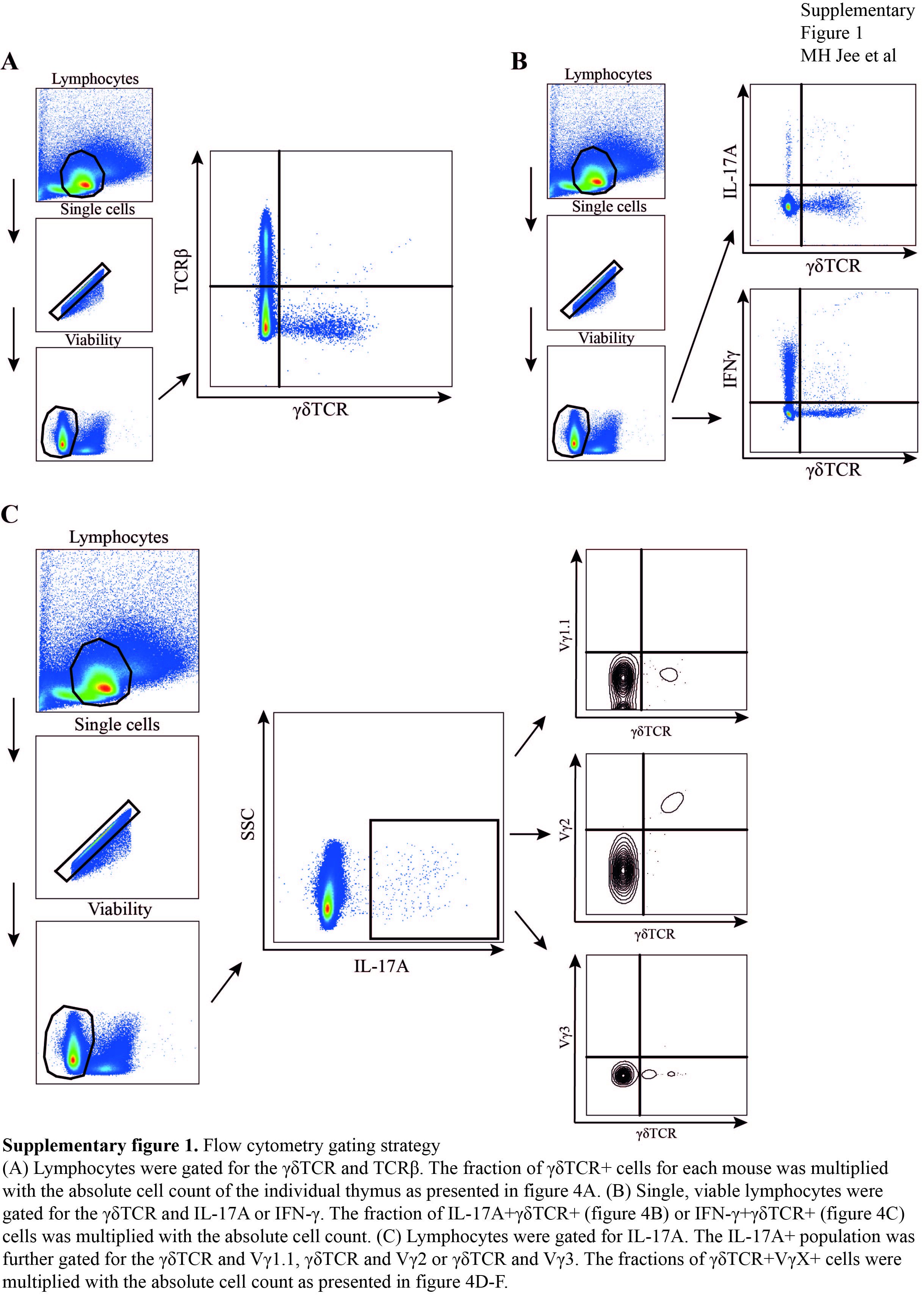

Supplement: Supplementary file 1 [file Image_1.jpg]

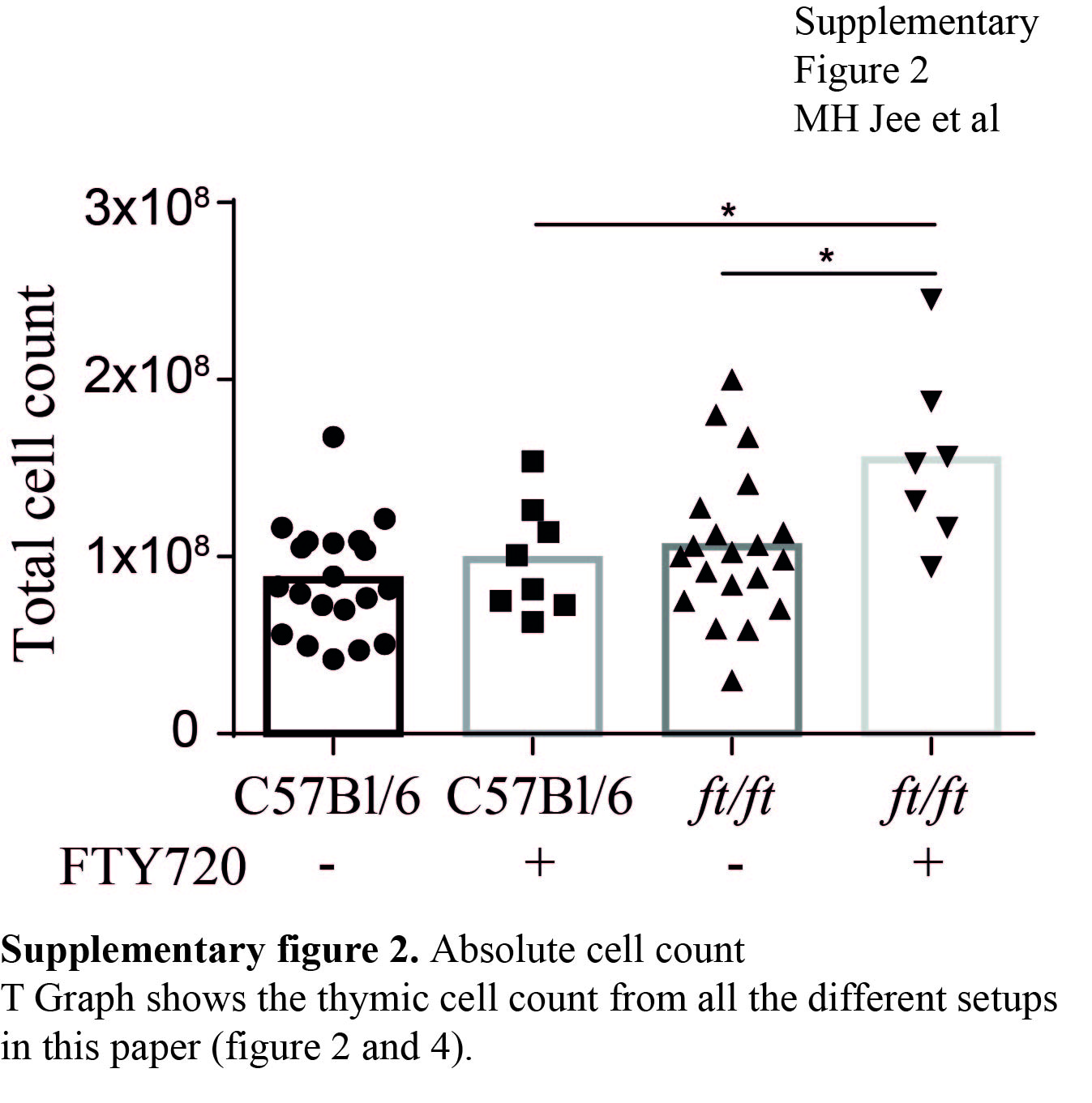

Supplement: Supplementary file 2 [file Image_2.jpg]

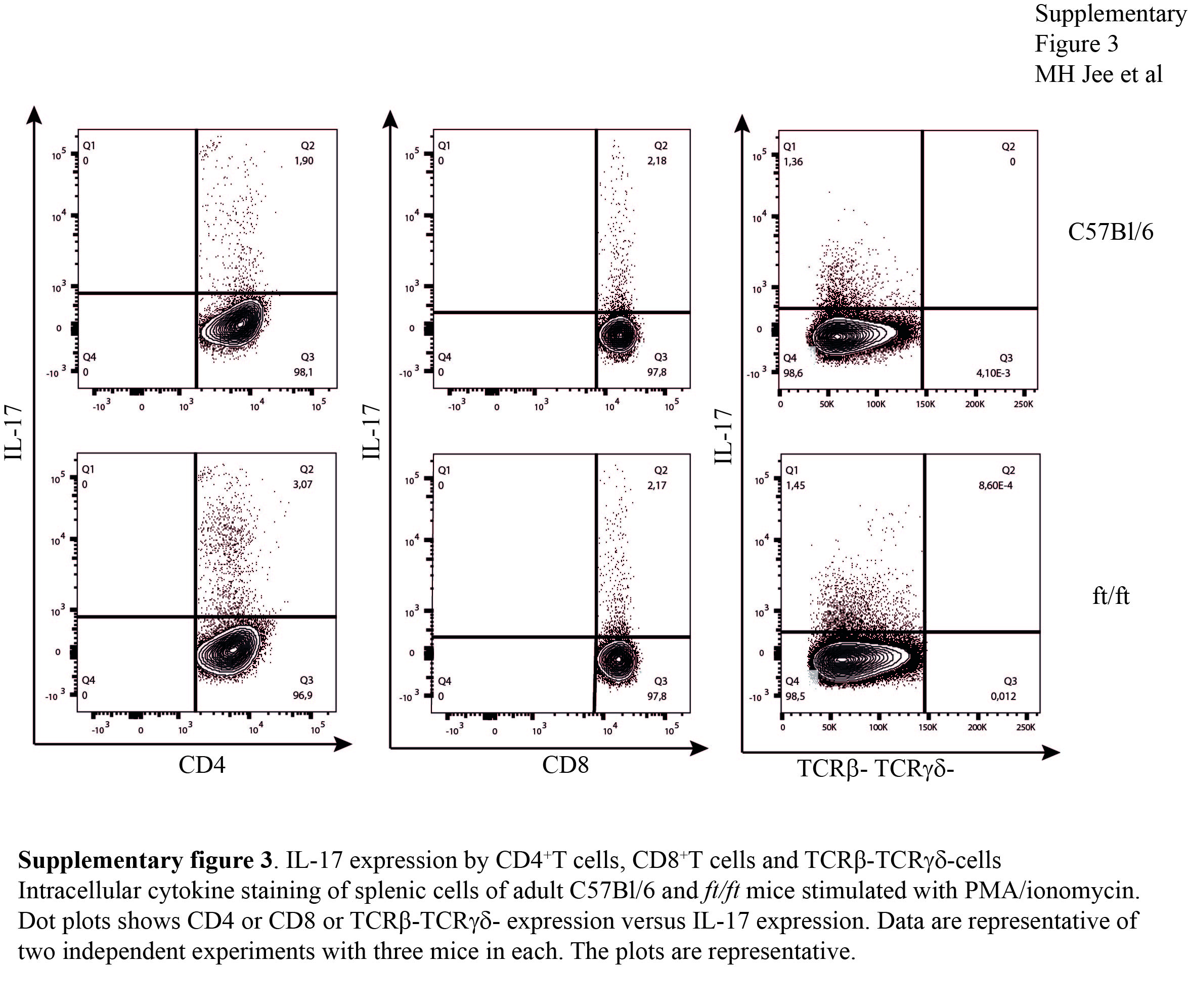

Supplement: Supplementary file 3 [file Image_3.jpg]
